# Investigation on the Exit Burr Formation in Micro Milling

**DOI:** 10.3390/mi12080952

**Published:** 2021-08-12

**Authors:** Zhongwei Chen, Xian Wu, Kai Zeng, Jianyun Shen, Feng Jiang, Zhongyuan Liu, Wenjun Luo

**Affiliations:** 1 College of Mechanical Engineering and Automation, Huaqiao University, Xiamen 361021, China; czw18559232902@163.com (Z.C.); zk20013080003@163.com (K.Z.); jianyun@hqu.edu.cn (J.S.); 2 Institute of Manufacturing Engineering, Huaqiao University, Xiamen 361021, China; jiangfeng@hqu.edu.cn; 3 Xiamen Xiazhi Technology Tool Co., Ltd., Xiamen 361115, China; 13824336804@163.com (Z.L.); lwj459613939@yeah.net (W.L.)

**Keywords:** micro milling, exit burr formation, burr width, burr shape

## Abstract

The burr on micro part has harmful effect on the dimensional accuracy and service performance. The original control of exit burr formation during micro milling is desirable and advisable. In this paper, the formation mechanism of exit burr was studied based on the varying cutting direction during micro milling. Three exit burr control strategies were concluded, the material properties embrittlement, the support stiffness increasing and machining parameter optimizing operations. Then, micro milling experiments were carried out to investigate the exit burr morphology and size. It was found that the exit burr formation was attributed to the change of material flowing path at the exit surface, which was caused by the negative shear deformation zone that was induced by the discontinuous shape features. Different exit burr morphologies were classified; the triangle exit burr type was caused by the varying exit burr growing direction along the exit surface. The optimal machining parameters in micro milling to obtain a small exit burr were suggested.

## 1. Introduction

Recently, the increasing requirement for micro equipment greatly promoted the innovation and development of micro machining technology [1,2]. Micro milling is one of the commonly used operations to produce micro parts with small size and complex shape characters. Comparison with other micro machining operations, micro milling exhibits many excellent advantages which include good machining efficiency, low production cost and high machining precision [3,4]. In terms of machining principle, micro milling is the downscaling of tool diameter and machining parameters from macro milling. However, the cutting thickness is comparable with tool cutting edge radius and grain size of workpiece material during micro milling process. Some new issues have appeared in micro milling, which include the size effect, the minimum uncut chip thickness and ploughing effect [5,6]. These issues usually result in bad surface quality and serious burr during micro milling. The small burr that formed on micro milled parts has a harmful influence on the dimensional accuracy and service performance [7,8].

Subsequent deburring operations are usually needed to remove the burr [9]. Abrasive assisted brushing was employed to deburr on micro milled groove by George et al. [10]. Kumar et al. [11] reported the ultrasonic assisted abrasive deburring method, and their results showed a burr reduction of 92% was achieved. Intelligent robot technology also was popularly applied in deburring operations [12]. However, it was found that although the burr can be removed with deburring operations, some harmful effect may be introduced at the same time, such as production cost increasing and dimensional accuracy damage. Muhammad et al. [13] reported the deburring operations accounts for 30% of the total manufacturing cost. Hence, the original control of burr formation during machining process is desirable and advisable.

According to the position on workpiece, the burr can be classified into four types: entrance burr, side burr, top burr and exit burr. The exit burr is located on the exit surface position of workpiece, which exhibits large size and great effect on micro part quality. Chern [14] analyzed the burr formation mechanisms near the exit of orthogonal cutting; it was found that the exit burr was attributed to the negative deformation plane that is formed when the steady-state chip formation stops as tool approaches the end of cut. Ko et al. [15] reported that the exit burr formation mechanism during orthogonal cutting was divided into three steps, initiation, development and formation, and the quantitative prediction model of exit burr formation was proposed. Niknam et al. [16] studied the correlation between friction angle and exit burr during slot milling of aluminum alloys; it was found that the larger friction angle was accompanied by the decreasing exit up milling side burr and the increasing exit bottom burr. Lekkala et al. [17] studied the effects of machining parameters, which include the cutting speed, feed rate, cutting depth and tool diameter, on the exit burr in micro milling, the results showed that tool diameter played the most significant effect. Pankaj et al. [18] established a theoretical prediction model for exit burr height based on the simplification into orthogonal cutting, and the results showed error less than 10% compared with micro milling experiment on titanium alloy. Zhang et al. [19] proposed an analytical model to predict the height and width of exit burr. The error was found to limit to 16% with micro milling results. The exit burr formation is affected by many factors, such as material properties, tool geometries and machining parameters [20]. Most of these researches are mainly based on the simplification model of milling process into orthogonal cutting with the constant cutting direction and cutting thickness. However, the cutting direction and cutting thickness are continuously varying during micro milling. Thus, the exit burr formation in micro milling is more complex and maybe different to orthogonal cutting. The essential mechanism researches of exit burr formation during micro milling are still insufficient.

In this paper, the formation mechanism of exit burr at the exit edge during micro milling were analyzed based on the continuous variation of cutting direction. According to formation mechanism, some exit burr control strategies were concluded. Then, the effect of machining parameters on exit burr morphology and width was studied in micro milling experiments. The optimal machining parameters in micro milling to achieve a small exit burr were obtained.

## 2. Experimental Setup

**Figure 1:**
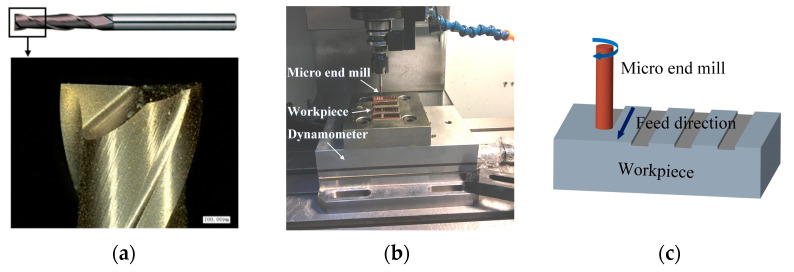
Micro end mill and micro milling experiment. (**a**) Micro end mill. (**b**) Micro milling experiment. (**c**) Schematic of full slot milling.

The used workpiece material was pure copper which is widely used for electrode material because of its excellent conductivity. The burr problem on copper electrode may cause some harmful effects in the electrical discharge machining process, such as the cavity size error and the poor product appearance. The coated micro end mill with two flutes was used in this work, which exhibits good cutting performance [21,22]. The tool diameter and helix angle were 1 mm and 30°, respectively, as shown in Figure 1a. The tool cutting edge radius and tool tip radius inspected with the optical microscope (VHX1000, Keyence, Osaka, Japan) were about 4.4 μm and 5.3 μm, respectively. Micro milling experiment was conducted using a compact vertical machining center (OM-2A, HAAS, Oxnard, CA, USA) which can provide the maximum spindle rotating speed of 30,000 rpm, as shown in Figure 1b. The workpiece was machined into small pieces using wire electrical discharge machining (WEDM, Hengtian, Taizhou, China) and then clamped on the worktable of machine tool. The workpiece was pre-machined to ensure flatness before experiments, and then the full slot milling was carried out, as shown in Figure 1c. The milling width was equal to the tool diameter of 1 mm. The machining parameters are listed in Table 1; the spindle speed was set from 8000 rpm to 20,000 rpm. The milling depth was set from 1 μm to 20 μm, to include the conditions that be larger and smaller than tool tip radius, and different levels of feed per tooth were set to include the conditions that they be larger and smaller than tool cutting edge radius.

**Figure 2:**
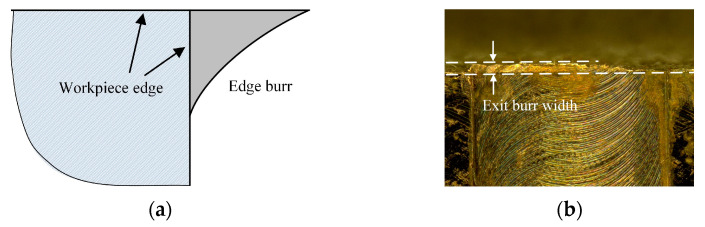
Exit burr definition and width measurement. (**a**) Exit burr definition. (**b**) Exit burr width measurement.

How to evaluate and measure of burr feature with small size in micro machining is a challenging and important task for mechanism research and parameter optimization. At present, although many researchers have presented a lot of studies on burr, there is no independent standard for the evaluation and measurement of burr features. The burr features often are included in the machining edge quality of workpiece. In ISO 13,715 for the definition of workpiece edge quality, the edge burr was defined as the residual material which overhangs outside the workpiece edge [23], as shown in Figure 2a. Based on this definition, the burr width and height are the common used indexes to evaluate the burr features in most literatures [24]. In addition, the burr area also is used to evaluate the burr features, but the measurement is very difficult [25]. Faith et al. compared the automated imaging process technique and the manual method to measure the burr width in micro milling, and they found that the imaging process technique presented a good measure accuracy [26]. Among three parameters, the burr width is relatively easy to measure by the optical microscope (VHX1000, Keyence, Osaka, Japan) and was employed to evaluate the exit burr features in this work. As shown in Figure 2b, the exit burr width was defined as the extension distance from the edge point to burr vertex point in the perpendicular direction of the exit surface. To measure the exit burr width, two lines parallel to the workpiece exit surface were drawn to envelop exit burr, and their perpendicular distance was considered as the exit burr width. To decrease error, the experiments has been repeated three times, and the averaged exit burr width was adopted as result.

## 3. Exit Burr Formation Mechanism

### 3.1. Exit Burr Formation Mechanism

**Figure 3:**
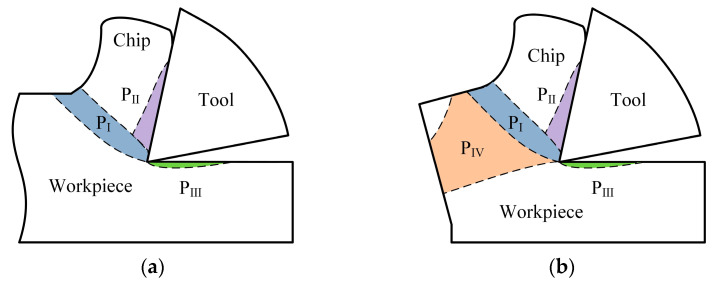
Schematic of negative shear deformation zone. (**a**) Three basic deformation zones. (**b**) The fourth deformation zone.

During the general micro milling process, the cutting region is composed of three deformation zones, i.e., the primary deformation zone (P_I_), the secondary deformation zone (P_II_) and the tertiary deformation zone (P_III_), as shown in Figure 3a. If the workpiece shape has continuous feature at the middle position, in the cutting region, the support stiffness in the front of cutting direction is very strong and presents very large resistance for material plastic flowing in this direction, but the upward direction is a free surface with minor resistance. Therefore, under the action of cutting force, the workpiece material plastic deformation happens at the (P_I_) zone and plastically flows in upward direction along the tool rake face to form chips. However, once the tool moves to near the exit surface of workpiece, the shape feature of workpiece changes to become discontinuous in the cutting direction. It leads to the suddenly decreasing support stiffness of workpiece in the front of cutting direction. In addition, the resistance to prevent material plastic flowing in this direction substantially weakens as well. This results in the exit surface of workpiece also becoming an unresisted free surface, and negative shear deformation zone (P_IV_) is produced underlying workpiece surface, as shown in Figure 3b. The direction of shear deformation in this zone is opposite to the primary deformation zone (P_I_). The appearance of negative shear deformation zone can change the material plastic flowing path in cutting process. The negative shear deformation zone extends to the exit surface of workpiece and has an intersection point with it. With the tool advancing, the intersection point acts as a plastic hinge. The material in negative shear deformation zone will no longer plastically flow in the upward direction but rotationally flow around the intersection point in the downward direction. The material above negative shear deformation zone just rigidly rotates around the intersection point almost without plastic deformation. The change of material plastic flowing path can generate an undesirable residual material that overhangs outside the exit surface on workpiece and forms the exit burr which is common burr type after cutting operation.

**Figure 4:**
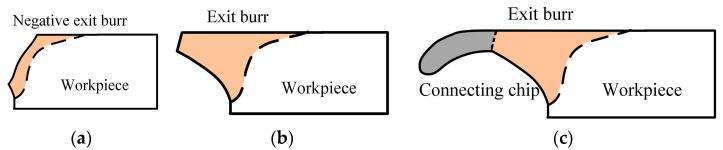
Three different cases of exit burr formation. (**a**) Negative burr. (**b**) Common exit chip. (**c**) Chip connecting with exit burr.

According to the fracture or not of exit burr, the shape characteristics of exit burr can be classified into three different cases. Once the tool moves to leave the exit surface, the stress in negative shear deformation zone gradually increases. In the first case, the stress value early exceeds the material fracture strength before the tool completely leaves the workpiece, the fracture occurs at the root of exit burr along the negative shear deformation zone, as shown in Figure 4a. This case can halt the subsequent burr formation process and produce the negative burr shape which is similar to a chamfering at the exit surface. It is common to observe as the workpiece materials present poor ductility. In the second case, the stress value in the negative shear deformation zone does not reach material fracture strength in the exit burr formation process, so at the exit burr, fracture does not occur, as shown in Figure 4b. The formed exit burr extends in the cutting direction and overhangs outside the exit surface of workpiece. This is the common shape characteristics of exit burr for plastic materials, such as copper material. In the third case, the chips do not separate in the primary deformation zone and connect with the end of exit burr after micro milling, as shown in Figure 4c. This case usually generates the large burr size.

**Figure 5:**
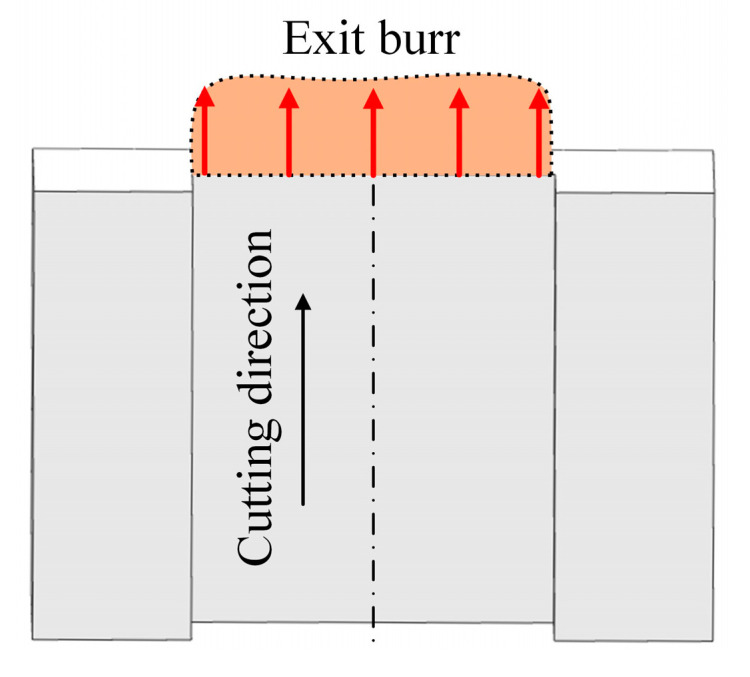
Exit burr growing direction in orthogonal cutting.

During orthogonal cutting, the tool directly cuts away from the whole exit surface of workpiece in only one time of cutting pass. The cutting direction and cutting thickness along the exit surface are constant. The exit burr always grows in the cutting direction during cutting process. Therefore, the growing direction of exit burr at the exit boundary is perpendicular to the exit surface in orthogonal cutting process, as shown in Figure 5. The shape morphology and size of the produced exit burr usually are relatively even at the exit surface.

**Figure 6:**
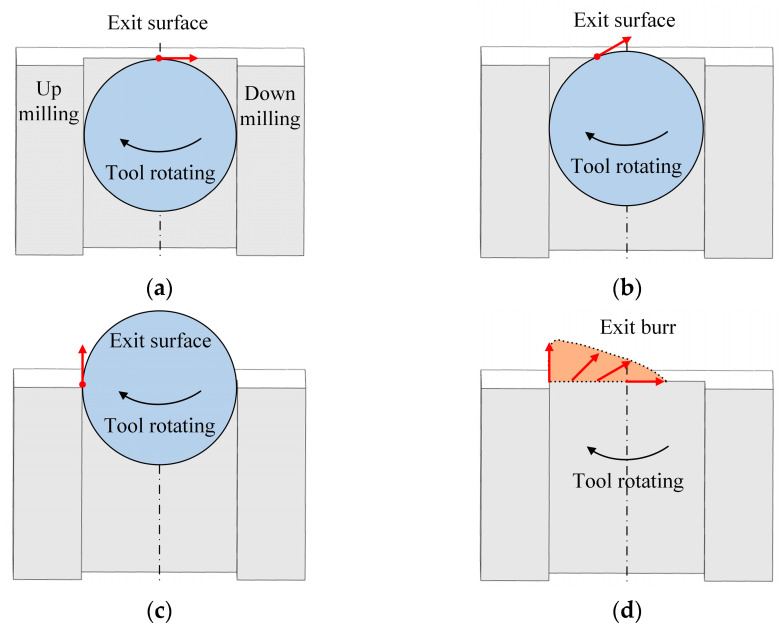
Exit burr growing direction in micro milling. (**a**) Parallel with exit surface. (**b**) Intersect with exit surface. (**c**) Perpendicular to exit surface. (**d**) Exit burr growing in micro milling.

However, in micro milling, the cutting path of each cutting edge is a circular, each cutting edge cut in workpiece at the up milling side and cut out workpiece at the down milling side. The rotating tool gradually cuts away from the exit surface of workpiece in serval times of cutting pass. The total number of cutting passes when the tool completely cuts out is related to the tool diameter and feed per tooth. When the tool advances to just arrive at the exit surface and the circular cutting path is tangent to the exit surface at the middle point, the tool begins to cut out from the workpiece, as shown in Figure 6a. At the tangent point, the cutting direction and exit burr growing direction are parallel to the exit surface and accompanied by the large cutting thickness which is equal to the feed per tooth. With tool advancing, when a small part of the tool has cut out from the exit surface, the circular cutting path intersects with the exit surface, as shown in Figure 6b. The intersection point is at the cutting out point and the cutting direction, and the exit burr growing direction forms an acute angle (0–90 °) with the exit surface. Simultaneously, the cutting thickness at the cutting out point gradually decreases. Once a half circle of tool has cut out from the exit surface, the circle center of cutting path is coincidence with the exit surface, as shown in Figure 6c. The up milling side point is the cutting out point, the cutting direction and the exit burr growing direction changes to perpendicular to the exit surface. The cutting thickness at the cutting out point becomes the smallest. It is found that with the cutting out point moving from the middle point to the side point along the exit surface, the cutting thickness and the exit burr growing direction are always continuously varying, as shown in Figure 6d. This is the significant difference in exit burr formation between the orthogonal cutting and micro milling. It can lead to the different shape and size features of exit burr in micro milling compared to orthogonal cutting. The exit burr formation in micro milling is more complex than orthogonal cutting.

### 3.2. Exit Burr Control Strategies

**Figure 7:**
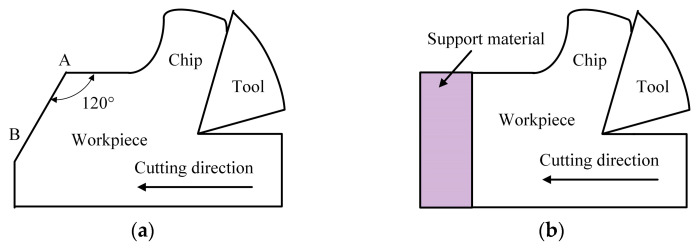
Support stiffness increasing methods. (**a**) Exit surface angle increasing. (**b**) Support material.

Based on the exit burr formation mechanism, it is concluded that the exit burr is produced by the unexpected material plastic flowing in negative shear deformation zone because of the support stiffness loss at the exit surface with the discontinuous shape feature. The exit burr size mainly is proportional to the amount of unexpected material plastic side flowing, which is depended on the material properties and stress distribution in the cutting zone. Hence, the following exit burr control strategies are suggested:

1. Material properties embrittlement. The metal materials with the higher plasticity and ductility usually produce the more unexpected material plastic flowing with the same stress distribution acting on it, and then, the larger exit burr will be produced. Therefore, the surface hardening treatment methods, which can increase the material brittleness and reduce the material ductility, are helpful to reduce the exit burr formation, such as the surface hammer peening and shot peening method. However, these material treatment methods need the additional operations and the increasing cost. Additionally, it is difficult to operate with micro part of a very small size.

2. Support stiffness increasing. The support stiffness loss at the exit surface is mainly responsible for the generation of the negative shear deformation plan. The auxiliary support at the exit surface which is an unresisted free surface can enhance the support stiffness in front of cutting direction and increase the resistance to prevent material plastic flowing in this direction. This leads to the small negative shear deformation zone (P_IV_) and the less exit burr formation. Hence, the support stiffness increasing methods can be used to control the exit burr in micro milling. As shown in Figure 7a, the exit surface angle between the exit surface and cutting direction is increased from 90° to 120°. With this structure design of exit surface, the slope surface AB plays the role of auxiliary support to increase the support stiffness of exit surface. This design is helpful to reduce the exit burr [27]. As shown in Figure 7b, the support material is fixed to close with the exit surface. The support stiffness at the exit surface of workpiece in the cutting direction is enhanced [28]. The exit burr will be formed on the exit surface of the support material instead of the workpiece. Then, the exit burr on workpiece can be effectively controlled.

3. Machining parameter optimizing. The amount of unexpected material plastic flowing in the negative shear deformation zone is proportional to the stress distribution. The tool geometry, cutting path and machining parameter have important effect on the stress distribution in micro milling process. The tool cutting edge radius is comparable to the cutting thickness in micro milling. The negative effective rake angle in cutting process will promote the formation of negative shear deformation zone at the exit surface and increase the exit burr. Therefore, it is helpful to reduce exit burr formation with machining parameters optimizing to weaken the negative effective rake angle. Machining parameters optimizing usually is the preferred choice to control exit burr because of its economical and effective characteristics without the additional process and cost increase. Hence, micro milling experiment was carried out to study the effect of machining parameters on the exit burr shape and size to obtain the guideline of machining parameter optimizing.

## 4. Experiment Results and Discussions

### 4.1. Exit Burr Morphology Characteristics

**Figure 8:**
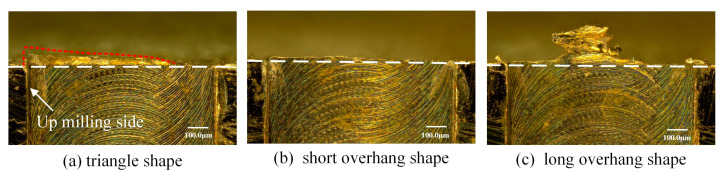
The exit burr shape classification. (**a**) Triangle shape. (**b**) Short overhang shape. (**c**) Long overhang shape.

The observed morphology characteristics of exit burr are shown in Figure 8. Different to orthogonal cutting, the exit burr morphology at the exit surface is very uneven in micro milling. It is found that the exit burr shapes can be classified into three different types, the triangle shape, the short and long overhang shape type. Among them, the triangle shape burr shows the medium size. The base of the triangle shape is the exit surface of workpiece, and the height of triangle burr gradually decreases from the up milling side to the middle point on the exit surface, as shown in Figure 8a. The short overhang shape only has the root part with relatively even morphology, and shows the smallest size. The long overhang shape which is mainly distributed on the middle position of exit surface has two parts, the root part and the large overhang part with wrinkle features, as shown in Figure 8c. The long overhang exit burr exhibits the largest size. The negative burr shape that shown in Figure 4a is not observed as copper is a plastic metal material. Based on the exit burr morphology, it is indicated that the triangle shape and short overhang shape belong to the case that shown in Figure 4b. The long overhang shape belongs to the case that shown in Figure 4c, and is mainly composed of the exit burr and the connecting chip that does not separate with exit edge. It is obvious that the long overhang shape presents the worst effect on the edge quality and is the least expected burr shape due to its large size.

**Figure 9:**
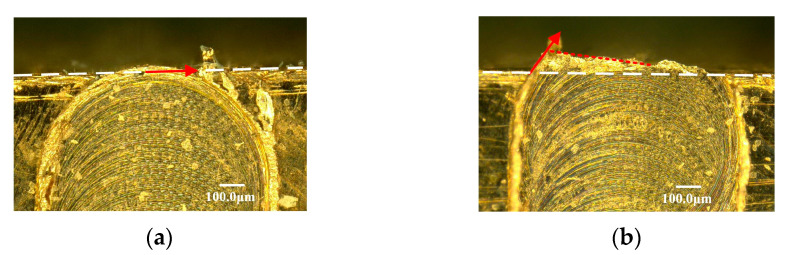
Exit burr formation process in micro milling. (**a**) Cut out at the middle point. (**b**) Cut out point move to side point.

The triangle shape of exit burr is caused by the continuous variation in the exit burr growing direction from the middle point to the side point along the exit surface. When the tool cutting edge cut out workpiece at the middle point of exit surface, the exit burr growing direction is parallel to the exit surface, as shown in Figure 9a. It leads to the small size of exit burr in the perpendicular direction of the exit surface at this cutting out point. Once the cutting out point gradually moves to the up milling side, the exit burr growing direction gradually deflects to the perpendicular direction of the exit surface, as shown in Figure 9b. It is clear to observe the sector distribution of the cut out marks at the zone of up milling side. Consequently, the exit burr height in the perpendicular direction of the exit surface is increasing. Additionally, the small cutting thickness at the side point will be less than the minimum cutting thickness. The induced severe ploughing effect may produce the large exit burr at the side point as well. This reason results in the exit burr height in the perpendicular direction of the exit surface gradually increases from the middle point to the side point, and the triangle exit burr is formed.

**Figure 10:**
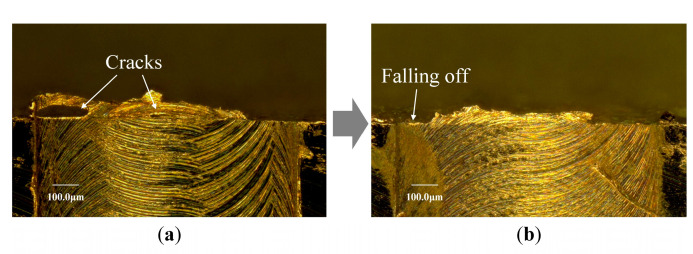
The tearing and falling of exit burr. (**a**) Micro cracks in exit burr. (**b**) Falling of exit burr.

It is found that some exit burrs exhibit incomplete shape along the exit surface, as shown in Figure 10. Due to the tool gradually cutting out from the exit surface in serval times of cutting pass, the exit burr which is formed at the previous cutting pass will suffer severe extrusion by tool bottom surface at the subsequent cutting pass. The overhanging exit burr is similar to a cantilever beam. It undergoes the largest bending moment at the root point. This weakest position easily generates micro cracks because of the severe extrusion in the subsequent cutting pass. The cracks will gradually propagate, resulting in the local tearing of exit burr, as shown in Figure 10a. Finally, the local tearing gradually evolves into the local fracture and falling of exit burr, as shown in Figure 10b. The rotating tool exhibits the largest cutting speed at the side point with the largest diameter, and produce the most severe extrusion on the previous exit burr. Hence, the tearing and falling of exit burr usually is firstly occurred at the side point. This reason explains the incomplete shape of exit burr which only remains part exit burr at the middle position of exit surface, as shown in Figure 8c. If the whole exit burr is falling off along the exit surface, the short overhang shape of exit burr that shown in Figure 8b is formed.

### 4.2. Exit Burr Size

**Figure 11:**
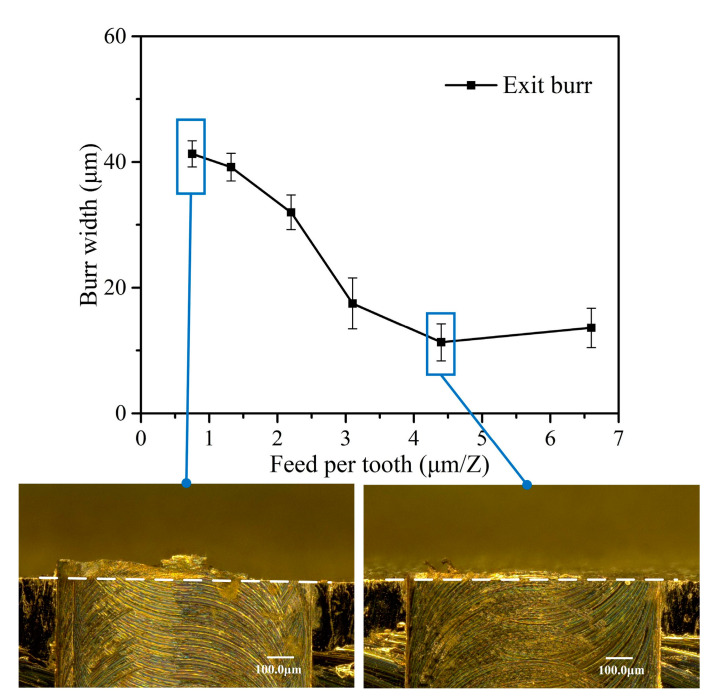
Exit burr shape and width vary with feed per tooth.

The exit burr morphology and size vary with the feed per tooth when *a_p_* = 10 μm and *n* = 16,000 rpm are shown in Figure 11. It is found that when the feed per tooth is less than tool cutting edge radius, the exit burr width rapidly increases with the decrease of feed rate. In this cutting condition, only the tool cutting edge arc participates in the cutting process owing to the small cutting thickness. The effective rake angle is actually negative and will become more negative with the further decrease of feed per tooth. The cutting condition with the negative effective rake angle usually generates the severe ploughing effect in the cutting process. The ploughing effect promotes material plastic deformation in the negative shear deformation zone (P_IV_) at the exit surface. This leads to the larger exit burr formation. The largest exit burr width is 41.3 μm obtained with *f_z_* = 0.75 μm/Z. When the feed per tooth is larger than tool cutting edge radius, the negative effective rake angle and ploughing effect are weakened owing to the increasing cutting thickness. Benefitting from the weakened material plastic deformation in the negative shear deformation zone (P_IV_), the exit burr width becomes stable at the relatively low level and shows little increment with the increase of feed per tooth. The smallest exit burr of 11.3 μm is achieved as the feed per tooth equal to tool cutting edge radius.

**Figure 12:**
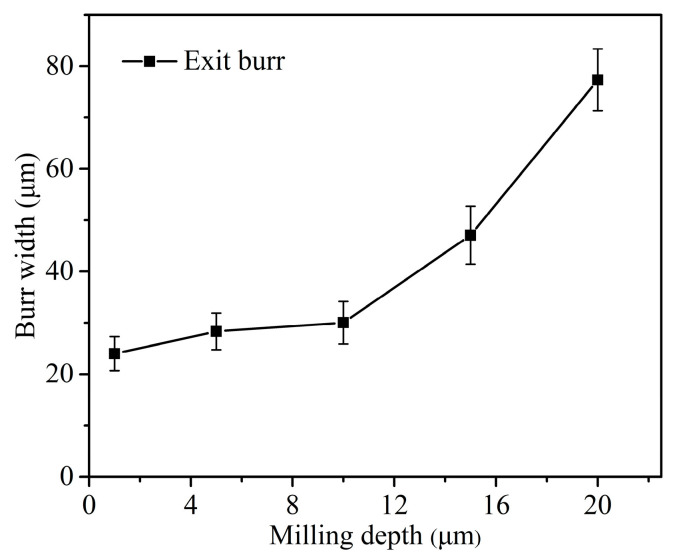
Exit burr width curve versus milling depth.

Figure 12 shows the exit burr size under different milling depth when *f_z_* = 3.1 μm/Z and *n* = 16,000 rpm. It is found that the exit burr width is gradually decreasing with the decrease of milling depth. The reason maybe that the material removal amount in once cutting pass becomes less with the smaller milling depth, and then, the cutting process presents the lower cutting force. This helps to alleviate the stress distribution in the negative shear deformation zone. It leads to the smaller exit burr formation. However, once the milling depth decreases to become lower than the tool tip radius of 5.3 μm, the exit burr width seems to be stable and just exhibits little decrement. In this cutting condition, only the tool tip arc participates in the cutting process. It leads to the actual cutting thickness in the interference region between the tool tip corner and the workpiece becoming less than the nominal feed per tooth during micro milling. This may increase the ploughing effect and material plastic deformation in negative shear deformation zone owing to the decreasing cutting thickness.

**Figure 13:**
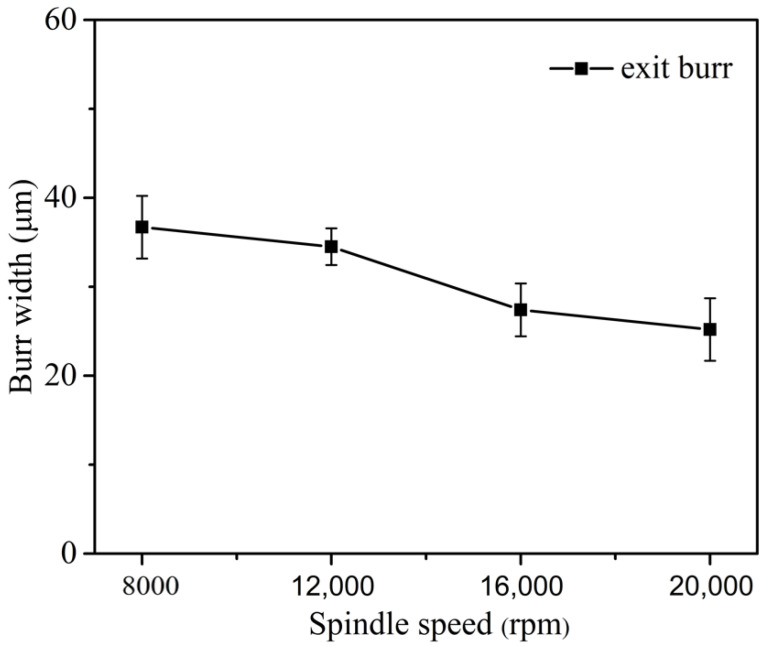
Exit burr width curve versus spindle speed.

Figure 13 shows the exit burr width under different spindle speed when *a_p_* = 10 μm and *f_z_* = 3.1 μm/Z. It is found that the exit burr width exhibits a gradually reduction trend with the increase of spindle speed. The tool can cut out from the workpiece in the shorter time when the cutting speed is increasing. Hence, the time for material plastic deformation in the negative shear deformation zone is reduced as well. Correspondingly, the relatively small exit burr formation is obtained, but compared with the feed per tooth and milling depth, the effect of spindle speed is relatively slight. Based on micro milling experiment results, it is concluded that the feed per tooth should be equal or larger than tool cutting edge radius, and the smaller milling depth and higher spindle speed also are helpful to achieve small exit burr in micro milling.

## 5. Conclusions

This paper presented an investigation on the exit burr formation mechanism during micro milling; the effect of machining parameters on exit burr morphology and width also was studied. Based on the results, the following conclusions are drawn:

1. When the tool advances to near the exit surface, the material plastic flowing path changes from the upward direction to the downward direction in the negative shear deformation zone which is induced by the sudden loss of support stiffness. The formation mechanism of exit burr is attributed to the change of material plastic flowing path at the exit surface. Different to orthogonal cutting, the cutting direction and the exit burr growing direction always vary at the exit surface. It leads to the different shape features of exit burr in micro milling compared to orthogonal cutting.

2. According to the exit burr formation mechanism, the exit burr size is proportional to the amount of unexpected material plastic flowing, which is depended on the material properties and stress distribution in the cutting process. Hence, three machining strategies to control the exit burr are suggested, i.e., the support stiffness increasing strategies at the exit surface, the material properties embrittlement operations at the exit surface and the machining parameter optimizing.

3. According to micro milling experiments, the triangle, the short and long overhang shape features of exit burr were observed. The triangle shape of exit burr is attributed to the varying exit burr growing direction and the decreasing cutting thickness from the middle point to the side point along the exit surface. The exit burr can obtain the smallest width since the feed per tooth equal to tool cutting edge radius. The exit burr width gradually decreases with the decrease of milling depth and the increase of spindle speed.

## Figures and Tables

**Table 1:** Micro milling parameters.

Parameters	Value
Spindle speed *n* (rpm)	8000, 12,000, 16,000, 20,000
Milling depth *a_p_* (μm)	1, 5, 10, 15, 20
Feed per tooth *f_z_* (μm/Z)	0.75, 1.32, 2.2, 3.1, 4.4, 6.6
